# Uptake of liquid from wet surfaces by the brush-tipped proboscis of a butterfly

**DOI:** 10.1038/srep06934

**Published:** 2014-11-06

**Authors:** Seung Chul Lee, Sang Joon Lee

**Affiliations:** 1 Department of Mechanical Engineering, Pohang University of Science and Technology, Pohang Gyeongbuk, Republic of Korea; 2 Center for Biofluid and Biomimic Research, Pohang University of Science and Technology, Pohang Gyeongbuk, Republic of Korea

## Abstract

This study investigated the effect of the brush-tipped proboscis of the Asian comma (*Polygonia c-aureum*) on wet-surface feeding. The tip region of this proboscis was observed, especially two microstructures; the intake slits through which liquid passes into the proboscis and the brush-like sensilla styloconica. The sensilla styloconica were connected laterally to the intake slits in the tip region. The liquid-feeding flow between the proboscis and the wet surface was measured by micro-particle image velocimetry. During liquid feeding, the sensilla styloconica region accumulates liquid by pinning the air-liquid interface to the tips of the sensilla styloconica, thus the intake slit region remains immersed. The film flow that passes through the sensilla styloconica region shows a parabolic velocity profile, and the corresponding flow rate is proportional to the cubed length of the sensilla styloconica. Based on these observations, we demonstrated that the sensilla styloconica promotes the uptake of liquid from wet surfaces. This study may inspire the development of a microfluidic device to collect liquid from moist substrates.

Small insects have been intensively studied recently in terms of their ability to transport liquid food to their mouthparts. These studies inspired the development of novel microfluidic techniques^1^. Butterflies are among the most common insects, and feed on liquid food through active suction^2^. In particular, they are generally known to feed on floral nectar, which they primarily drink through a long, coilable proboscis with a high pressure gradient induced by a cibarial pump^3^. Because of their importance as a pollinator, extensive investigations have been made into the functional anatomy of mouthparts^4^,^5^ and the relationship between intake rate and nectar concentration^2^,^6^,^7^,^8^. However, several members of the nymphalid butterfly feed exclusively on liquids from wet surfaces, such as rotten fruits, mud puddles, and tree sap^9^. These butterflies can uptake liquid more efficiently from wet surfaces than nectar-feeding butterflies can^10^, and they have brush-tipped proboscises composed of numerous elongated sensilla structures near the tip. Such structures are lacking or reduced in nectar-feeding butterflies^9^. Thus, the brush-tipped structure presumably plays a functional role in the uptake of liquid food from wet surfaces^10^,^11^. Lehnert et al.^11^ investigated the wetting characteristics of the proboscis and reported that the brush structure enhances wettability at the tip region. However, relatively little is known about how the brush-tipped proboscis promotes the efficiency of liquid uptake from wet surfaces.

In the current research, we study the nymphalid butterfly Asian comma (*Polygonia C-aureum*), which can feed on wet surfaces. The morphological structures of the proboscis tip region are observed using synchrotron X-ray micro-computed tomography (CT), and its functional characteristics are discussed in terms of interactions with wet surfaces. We also measure the uptake process of liquid from the wet surface through a micro-particle image velocimetry (PIV) under in vivo conditions to determine the effects of the brush-like structure sensilla styloconica on the uptake of liquid from wet surfaces. This study may be helpful to understand the structural adaptation of the proboscis to wet-surface feeding and inspire a microfluidic device for collecting liquid from moist substrates.

## Results

### Morphology of the proboscis

**Figure 1:**
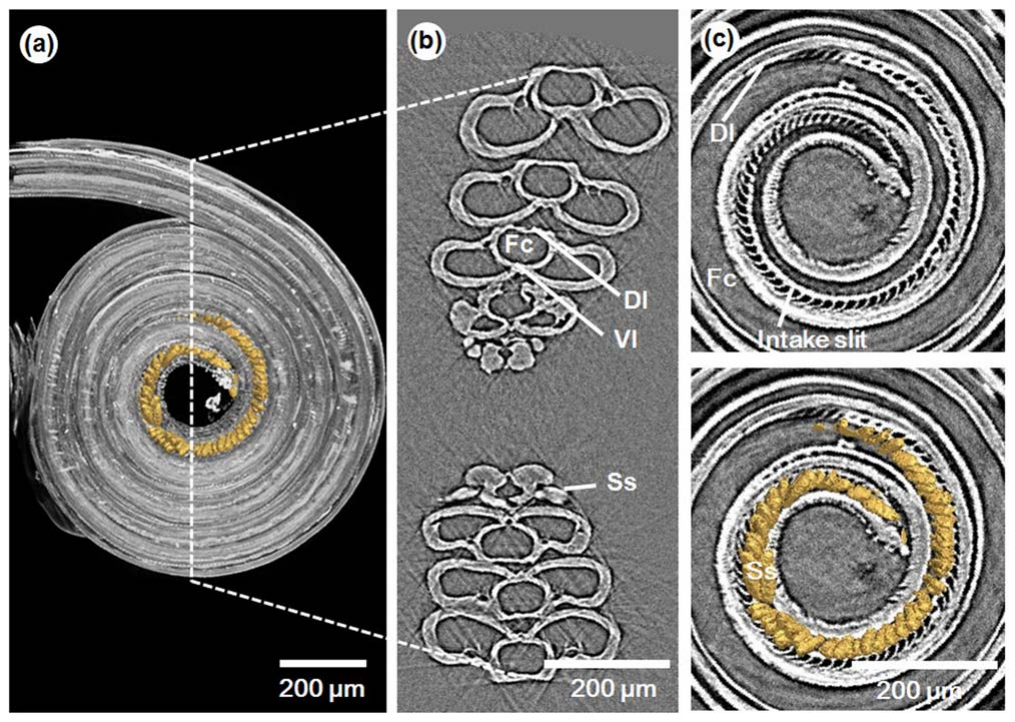
Morphological structure of the proboscis of the Asian comma (*Polygonia c-aureum*). (a) Coiled proboscis with sensilla styloconica (yellow) (b) Image of the cross-section that passes through the center of the coiled proboscis. *Fc*. food canal; *Dl*. dorsal linkage; *Vl*. ventral linkage; *Ss*. sensilla styloconica. (c) Sagittal images of the area near the tip. (Modification of the dorsal linkage coincides with the appearance of sensilla styloconica).

**Figure 2:**
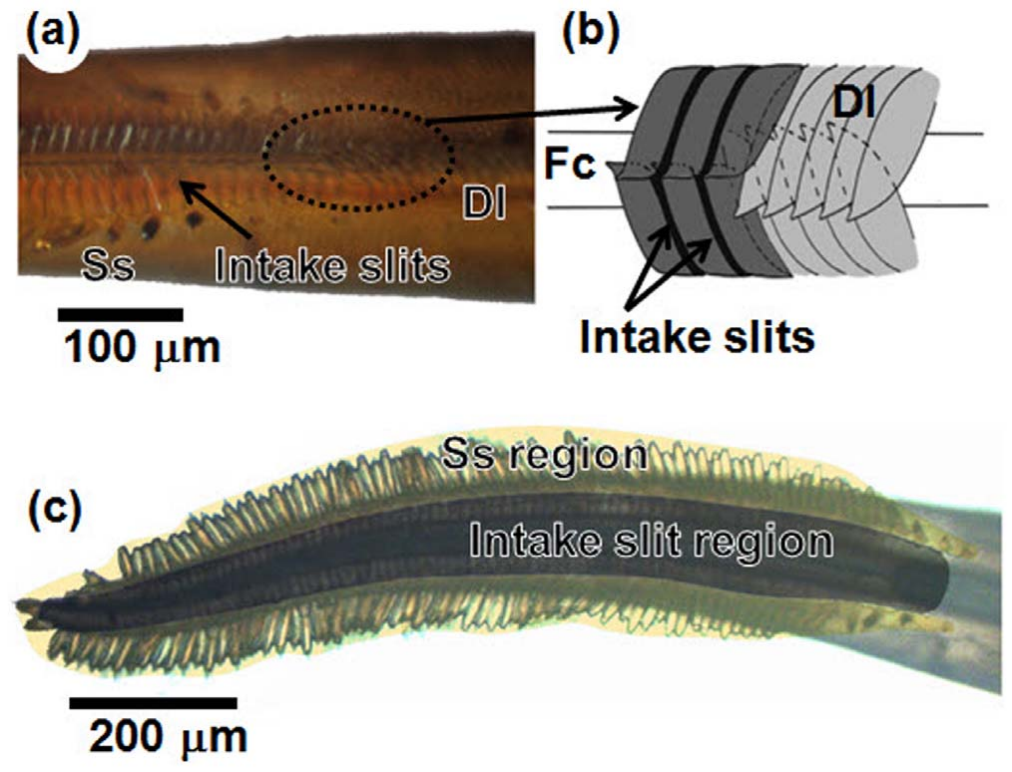
Tip region of the straightened proboscis. (a) Proximal end of the tip region at which the structure of the dorsal linkage begins to change. (b) Schematic of the dorsal linkage mechanism (not to scale). (c) Tip region is composed of two regions; intake slit and sensilla styloconica regions.

We identified the structural characteristics of the proboscis used to uptake liquid from wet surfaces through synchrotron X-ray micro-CT. 3D reconstruction images of the proboscis and its cross-sectional images were generated. The proboscis of the test butterfly was coiled (Fig. 1a); thus, several cross-sections of the proboscis were observed and their morphological characteristics were analyzed (Fig. 1b). The proboscis was composed of two galeae that were linked dorsally and ventrally to form a food canal between them^4^ (Fig. 1b). The widths of the two galeae and of the food canal were relatively constant, with the exception of the tapered tip region^12^. The decreased galea width in this region is offset by the elongated sensilla structure known as the sensilla styloconica, which is observed laterally (Fig. 1b). The overall external width of the proboscis is thus maintained. The cross-section of the proboscis is flat with a wide dorsal side as in Fig. 1b, and its aspect ratio is approximately 3.37:1. In addition to this wide cross-sectional area, the proboscis displays a projecting dorsal linkage structure (Fig. 1b). To determine the morphological characteristics of the tip region, the formations of the inlet structure and of sensilla styloconica were examined in detail. The sagittal cross-section images indicate that the dorsal linkage near the tip is shaped like a curved turbine blade (Fig. 1c). This morphological feature leaves intake slits between the curved linkage structures (Figs. 2a, b). The dorsal linkage structures overlap well and function as the roof wall of the food canal along the rest of the proboscis^4^ (Figs. 2a, b).

The sensilla styloconica appear at the tip region of the proboscis, where the dorsal linkage has intake slits (Figs. 1c, 2a). The tip region of the proboscis consists of the intake slit region and the brush-like sensilla styloconica region (Fig. 2c). Table 1 summarizes the physical dimensions of the proboscis, especially those of the tip region. The number of intake slits is slightly smaller than that of the sensilla styloconica. Furthermore, the width of the slit corresponds to almost one sensilla styloconica in the distal 80% of the tip region. In the proximal 20% of the tip region, the number of sensilla styloconica decreases. The distribution of the intake slit is nearly uniform along the tip region.

### In vivo measurement of the uptake process of liquid from the wet surface

We further investigate the liquid-feeding process involving the tip region of the proboscis and the wet surface. The test butterfly in this study was induced to drink liquid by covering the dorsal side of its straightened proboscis with a wet cover slip. The liquid uptake process between the proboscis and the cover slip was then measured with a micro-PIV velocity field measurement technique. The focal plane (measurement plane) was adjusted to include the contact surface between the proboscis tip region and the wet cover slip.

**Figure 3:**
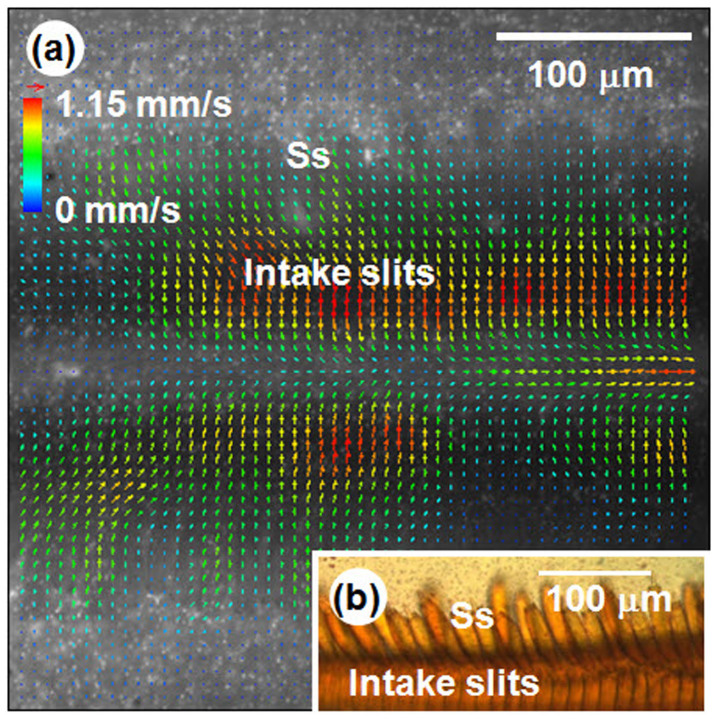
Flow velocity field at the tip region of the proboscis sucking water from the wet cover slip. The water was seeded with fluorescent trace particles. (a) 2D velocity field of the flow between the proboscis and the wet cover slip. (b) Microscopic image of the intake slits and the sensilla styloconica.

The liquid film that formed on the cover slip soaked the tip region of the proboscis, which was sucked by the test butterfly. The velocity of intake flow accelerated and decelerated periodically, reflecting the systaltic motion of the cibarial pump located in the head of the butterfly. The feeding frequency measured 3.5 ± 0.86 Hz (*N* = 3). The velocity vector field of the suction flow was analyzed at the fastest flow phase in one feeding cycle. This flow was accelerated toward the intake slit region of the proboscis and velocity was maximized in this region (Fig. 3a). The intake slit functioned as the inlet through which the uptaken liquid enters the food canal. The directions of the velocity vectors of suction flow are parallel to those of the sensilla styloconica (Fig. 3). The sensilla styloconica seems to guide the liquid to the intake slits, which implies that this structure may work as extended slits. The maximum velocity is approximately 1.15 mm/s, and the corresponding capillary number (Ca = *μV/γ*) is 1.60 × 10^−5^ based on the water properties (*μ* = 0.001 N·s/m^2^, *γ* = 0.072 N/m).

**Figure 4:**
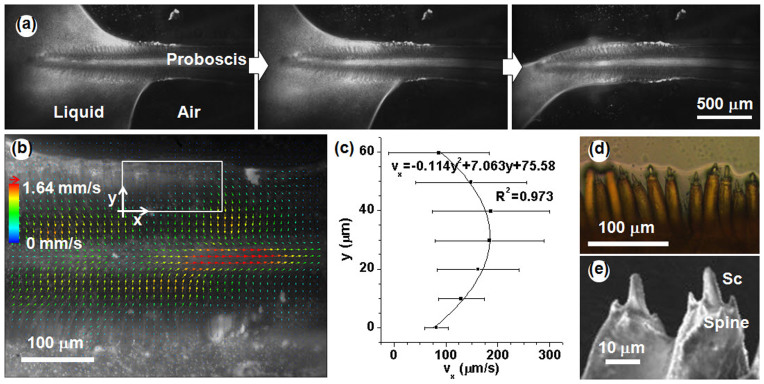
Interaction between the proboscis and the liquid on the wet surface. (a) Changes in the air-liquid interface during liquid-feeding process. (b) Flow velocity field of the liquid accumulated in the sensilla styloconica region. The white square indicates the coordinate system used in the velocity analysis. (c) Parabolic x-direction velocity profile of the flow in the sensilla styloconica region. (d) Air-liquid interface was supported by the tips of the sensilla styloconica. (e) SEM image of the tips of the sensilla styloconica. *Sc.* sensory cone.

We also examined the changes in the air-liquid interface during liquid-feeding process. The liquid on the wet cover slip was drained by the continuous feeding of the test butterfly, and the meniscus of the liquid receded gradually (Fig. 4a). However, the edge of the air-liquid interface on the proboscis side started to adhere to the tips of sensilla styloconica, and the liquid in the sensilla styloconica region did not disappear by suction (Figs. 4a, b). Where the tips of sensilla styloconica did not adhere to the air-liquid interface (liquid did not recede yet), the flow field showed the same pattern as previously depicted in Fig. 3. To examine the flow of the liquid trapped in the sensilla styloconica region, we extracted the *x*-direction (direction parallel to the proboscis length) velocity (*v_x_*) in the white square depicted in Fig. 4b. The *v_x_* were plotted against the sensilla styloconica length (y = 0 μm to 60 μm). The pattern of the velocity profile is similar to that of the parabolic flow between two plates (Fig. 4c). The bottom plate (*y* = 0) corresponds to the base of the sensilla styloconica that is connected to the intake slit region, and the upper plate is the air-liquid interface that is supported by the tips of the sensilla styloconica (Figs. 4b, d). This result shows that the liquid flows up through the sensilla styloconica region in the direction of proboscis length during liquid feeding. As a result, the entire intake slits in the tip region are immersed in liquid throughout liquid-feeding process. The liquid surrounding the tip of the proboscis is gradually reduced; consequently suction flow is accelerated. In the process, the maximum flow speed is increased (*V* = 1.64 mm/s). The Reynolds number (Re = *ρVe/μ*) based on the maximum flow velocity (*V*), the length of sensilla styloconica (*e* = 60 μm) and the water properties (*ρ* = 1000 kg/m^3^, *μ* = 0.001 N·s/m^2^) was much lower than 1 (Re = 0.0984).

To observe the maintenance of the accumulated liquid in the air-liquid interface in detail, we cut the straightened proboscis from the butterfly head and exposed it to a small-scale liquid droplet. The straightened proboscis was immediately soaked with the absorbed liquid through the sensilla styloconica structure. The sharp tips of the sensilla styloconica supported the air-liquid interface (Fig. 4d). The apical region of the sensilla styloconica exhibits a hierarchical sharp structure that consists of a central sensory cone surrounded by sharp spines (Fig. 4e).

### Comparison with a nectar-feeding butterfly

**Figure 5:**
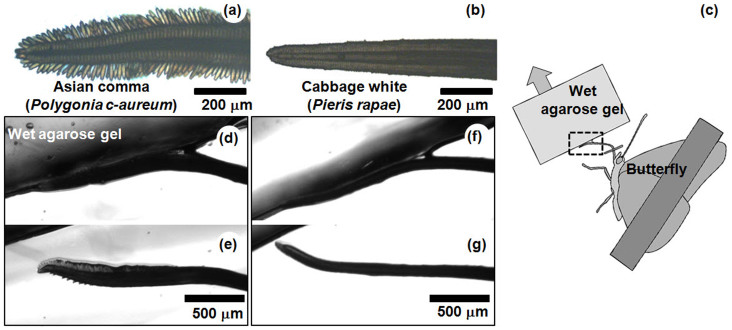
Difference in the liquid accumulation between wet-surface feeding and nectar-feeding butterflies. Tip regions of the proboscises of two butterflies: (a) Asian comma (*Polygonia c-aureum*) and (b) cabbage white (*Pieris rapae*). (c) Schematic of the experimental setup to observe the accumulation of liquid from the wet surface by the proboscis. (d–e) and (f–g) show the detachment of the proboscises of the Asian comma and the cabbage white from the wet agarose gel, respectively.

The liquid uptake of the test butterfly was compared to that of a typical nectar-feeding butterfly cabbage white (*Pieris rapae*) to determine the effects of the brush-shaped tip on liquid accumulation. The proboscis of a cabbage white does not have a brush-shaped tip; rather, it is pipet-like in appearance (Fig. 5b). The proboscises of both butterflies were similar in terms of morphological features, including linkage structures and intake slits^9^,^13^. Furthermore, the cibarial pumps of both butterflies possess nearly identical composition and mechanism^14^. The expansion of the cibarial pump creates negative pressure to imbibe liquid through a proboscis. Both butterflies were induced to touch the wet surface by placing the agarose gel soaked with 10% sucrose solution near the tip (Fig. 5c). The tip region was not seen when it adhered to the wet surface of the agarose gel (Figs. 5d, f). To observe the tip region, we then carefully detached the proboscis from the wet surface by moving the agarose gel. During detachment, the dorsal side of the proboscis began to adhere to the slide glass, which functioned as a sample stage. The dorsal views of the tip regions of the proboscises of both butterflies differed with respect to liquid accumulation. In the Asian comma, liquid was trapped in the sensilla styloconica region (Fig. 5e), whereas the proboscis tip region of the cabbage white could not retain liquid (Fig. 5g). In addition, we compared the liquid-feeding abilities of the two butterflies from the wet surface by measuring temporal variations of the area occupied by the liquid on the cover slip during liquid-feeding process (Fig. 4a). For the case of the cabbage white in this process, the liquid around the proboscis tip was also sucked into the intake slits. However, the proboscis could not continuously hold the liquid, because the contact of the intake slits with the liquid was gradually lost as the amount of liquid decreased. In this process, the maximum flow velocity was approximately 0.845 mm/s. The Reynolds number and capillary number in the cabbage white proboscis were 0.05 and 1.17 × 10^−5^, respectively. The areal liquid uptake rates of both the Asian comma and the cabbage white were approximately 4.54 × 10^−1^ mm^2^/s and 2.39 × 10^−1^ mm^2^/s, respectively. The areal uptake rate may represent the volume liquid uptake rate because both butterflies feed a sheet of liquid on the cover slip at the same condition. Thus, the liquid uptake rate of the Asian comma was approximately twice as fast as that of the cabbage white in wet-surface feeding.

## Discussion

Butterflies with brush-tipped proboscises sucked more liquid from wet surfaces than nectar-feeding butterflies did^10^; thus we experimentally demonstrated how a brush-tipped proboscis promotes liquid uptake from wet surfaces in current study.

**Figure 6:**
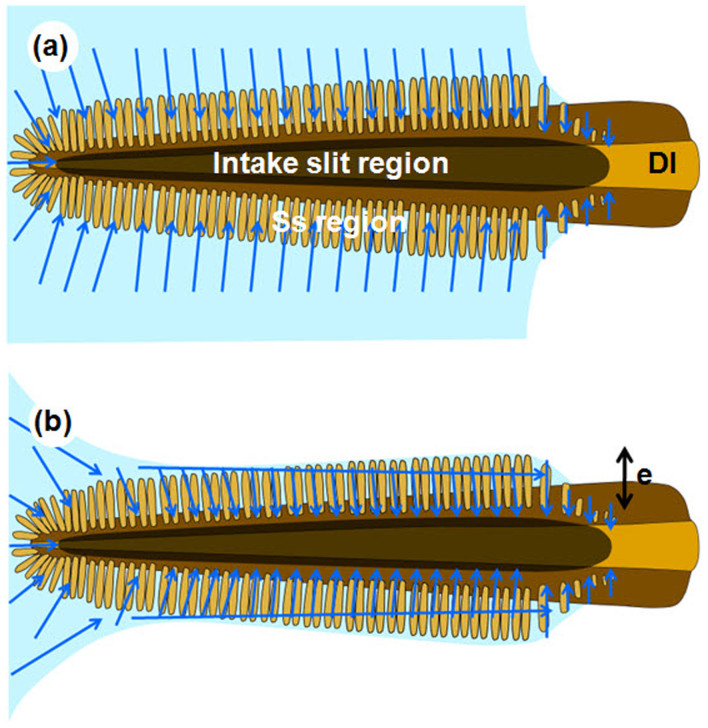
Model established for uptake of liquid from wet surfaces by a brush-tipped proboscis. The schematics represent the dorsal side of the tip region of the proboscis that touches the wet surface (not to scale). (a) When a butterfly touches the wet surface with the dorsal side of its proboscis, the intake slit region (dark gray) is immediately soaked with the aid of capillary force. The thin liquid layer (light blue) is sucked into the intake slits through the sensilla styloconica, which in turn collects water and expands the suction area. (b) Liquid is accumulated in the sensilla styloconica region while the intake slit region remains immersed to promote liquid intake. The blue arrows indicate the flow directions. *e* is the thickness of the accumulated liquid.

A wide inlet structure may facilitate the efficient suction of exposed liquid from large surfaces, such as rotting fruits, mud puddles, and tree saps. The intake slits allowing liquid to enter the food canal protrudes from the dorsal side to make contact with wet surfaces. The elongated sensilla styloconica were serially connected to the intake slits in 80% of the tip region. The flow to the intake slit was guided by the sensilla styloconica, which suggests that this structure extends the effective inlet area and increases the area of contact with wet surfaces. Moreover, both the sensilla styloconica and intake slits constitute parallel micro-grooves in the tip region. This micro-texture promotes capillary flow on the surface^15^. The small capillary number in the order of 10^−5^ indicates that capillary force would mainly contribute to the initial imbibition from wet surfaces although butterflies feed on liquid by active suction (Fig. 6a).

Liquid accumulation is another important function of the sensilla styloconica region. The intake slit region must remain in contact with the liquid for efficient liquid uptake^9^. The brush-like structure of the sensilla styloconica imbibes and traps liquid by pinning the air-liquid interface to their hierarchical sharp tips. Therefore, liquid is sustained around the tip region during liquid feeding. The accumulated liquid does not remain static but continuously enters the intake slit region through the sensilla styloconica region. The velocity of the flow that passes through this region in the direction of the proboscis length exhibits a parabolic profile (Figs. 4b, c). This parabolic viscous flow can be attributed to the small Reynolds number (Re ≪ 1). The flows measured at the upper and bottom boundaries (*y* = 0 μm, 60 μm) seem to slip possibly because the boundaries are not solid walls but are interfaces. The width of the accumulated liquid can be estimated to be equal to the length of sensilla styloconica (*e* = 60 μm; Fig. 6b). The volume of the flow rate (*q*) of the film flow that passes through the sensilla styloconica region was evaluated by integrating the parabolic velocity profile, which is proportional to *e*^3^. The brush-tipped proboscis of the Asian comma accumulates liquid from the wet agarose gel in the tip region, whereas the proboscis of the cabbage white cannot retain liquid because it does not have this brush-tipped structure. The accumulation of liquid in the sensilla styloconica region has an important role because the cabbage white also feeds liquid at similar Reynolds number and capillary number condition. Based on these observations, we suggested that the brush-tipped proboscis enable suck up more liquid (*q* ~ *e*^3^) than that without a brush-like structure during wet-surface feeding.

Krenn et al.^9^ reported that wet-surface feeding butterflies possess more and longer sensilla styloconica than nectar-feeding butterflies do. This morphological difference may explain the adaption of butterfly proboscises to wet-surface feeding. Proboscises with micro-textured tip region can enhance the capillary imbibitions of liquid^15^ and increase the areas of effective suction. The air-liquid interface of the accumulated liquid is stably supported by the numerous sensilla styloconica. The elongated sensilla styloconica enlarged the area for intake flow, which increases flow rate *q*. Finally, this study may be helpful to understand the liquid uptake behavior of butterflies in wet-surface feeding and to motivate the development of bio-inspired technology for the collection of liquid from moist substrates.

## Methods

### Butterfly and sample preparation

To investigate the mechanism of liquid intake from wet surfaces, the Asian comma (*Polygonia c-aureum*) was selected as a test sample. This butterfly can feed on liquid from wet surfaces such as rotting fruits. For comparison, a typical nectar-feeding butterfly, cabbage white (*Pieris rapae*) was selected. Both species were purchased from the Little Pet (Seoul, Republic of Korea). The test butterfly was immobilized by taping the folded wings to a slide glass. Its proboscis was carefully uncoiled using a glass micro-needle and then fixed with a small piece of tape (Fig. 5c).

### Observing liquid accumulation from wet surface

An agarose (Agarose-LE, Affymetrix Inc., CA, USA) gel block was soaked with 10% sucrose solution and placed near the tip region of the partially fixed proboscis (Fig. 5c). The butterfly touched the wet agarose gel with the dorsal side of its proboscis after a few seconds. The agarose gel was then carefully detached from the proboscis tip using micro-forceps. This process was recorded by an inverted microscope (Axiovert 200, Carl Zeiss, Oberkochen, Germany) equipped with a CCD camera (Sensicam, PCO, Kelheim, Germany).

### Morphological analysis of the proboscis

The morphological features of the straightened proboscis were observed using an optical microscope (Eclipse 80i, Nikon, Tokyo, Japan) attached with a digital camera (D700, Nikon, Tokyo, Japan). The cross-sectional slice images of the proboscis was obtained by the synchrotron X-ray micro-CT system in the 6C Biomedical Imaging (BMI) beamline of Pohang Accelerator Laboratory (PAL, Pohang, Republic of Korea). The 6C BMI beamline provided monochromatic X-ray beam raging from 10 keV to 60 keV. The proboscis was separated from the head of the living butterfly, and fixed onto a disposable pipet tip with instant glue. The test sample was then mounted on a rotating stage through which the X-ray beam passed. Total 361 X-ray projection images of the proboscis were acquired by a sCMOS camera (Zyla, Andor, Belfast, UK) at 0.5° intervals during rotation from 0° to 180°. The projection images were reconstructed using Octopus software (in CT, Ghent, Belgium). Moreover, the images were reconstructed in 3D with Amira software (Visualization Sciences Group, Burlington, MA, USA).

### Flow measurements

The interaction between the liquid from the wet surface and the proboscis tip was investigated through a micro-PIV technique. The dorsal side of the uncoiled proboscis on the slide glass was exposed to the wet surface by covering a cover slip wetted with water mixed with fluorescent tracer particles (mean diameter ~ 1.0 μm, Molecular Probes, Eugene, OR, USA). A continuous Nd: YAG laser (*λ* = 532 nm, SLOC, Shanghai, China) was used for volume illumination at the tip of the proboscis of the liquid-feeding butterfly. The fluorescent tracer particles emitted fluorescence at a wavelength of 554 nm after absorbing the laser light. The fluorescent particle images were selectively observed using a microscope (Eclipse 80i, Nikon, Tokyo, Japan) equipped with a long-pass filter (*λ* > 550 nm). They were recorded by a high-speed CMOS camera (Photron ultima APX, Fujimi, Tokyo, Japan) at a frame rate of 2,000. The velocity field of liquid uptake flow was obtained by applying a cross-correlation PIV algorithm to two successive fluorescent images.

## Author Contributions

S.C.L. designed and conducted the experiments, analyzed the data and wrote the manuscript; S.J.L. designed the experiments, analyzed the data and wrote the manuscript.

## Figures and Tables

**Table 1:** Dimensional specifications of the proboscis of the Asian comma (*N* = 5)

	Mean ± S.D.
Proboscis length [mm]	12.89 ± 0.95
Length of the intake slit region [mm]	1.269 ± 0.053
Number of intake slits per galea [N]	86.4 ± 5.8
Length of the sensilla styloconica region [mm]	1.290 ± 0.038
Number of sensilla styloconica per galea [N]	77.2 ± 6.6
Length of sensilla styloconica [μm]	60.35 ± 5.04
